# Protocol for Biomarker Ratio Imaging Microscopy with Specific Application to Ductal Carcinoma *In situ* of the Breast

**DOI:** 10.3389/fcell.2016.00120

**Published:** 2016-11-03

**Authors:** Andrea J. Clark, Howard R. Petty

**Affiliations:** Department of Ophthalmology and Visual Sciences, University of Michigan Medical SchoolAnn Arbor, MI, USA

**Keywords:** biomarkers, cancer, ductal carcinoma *in situ*, ratio imaging, microscopy, stem cells, mesenchymal cells

## Abstract

This protocol describes the methods and steps involved in performing biomarker ratio imaging microscopy (BRIM) using formalin fixed paraffin-embedded (FFPE) samples of human breast tissue. The technique is based on the acquisition of two fluorescence images of the same microscopic field using two biomarkers and immunohistochemical tools. The biomarkers are selected such that one biomarker correlates with breast cancer aggressiveness while the second biomarker anti-correlates with aggressiveness. When the former image is divided by the latter image, a computed ratio image is formed that reflects the aggressiveness of tumor cells while increasing contrast and eliminating path-length and other artifacts from the image. For example, the aggressiveness of epithelial cells may be assessed by computing ratio images of N-cadherin and E-cadherin images or CD44 and CD24 images, which specifically reflect the mesenchymal or stem cell nature of the constituent cells, respectively. This methodology is illustrated for tissue samples of ductal carcinoma *in situ* (DCIS) and invasive breast cancer. This tool should be useful in tissue studies of experimental cancer as well as the management of cancer patients.

## Introduction

The advent of novel screening methods have substantially reduced the incidence of advanced forms of colon and cervical cancers (Janicek and Averette, 2001; Pignone et al., 2002), as might be expected by removing patients with precursor lesions from the patient pool. Other screening assays have not had such compelling successes. For example, mammography has had only a minor effect on the prevalence of invasive breast cancer (Ozanne et al., 2011; Bleyer and Welch, 2012; Harding et al., 2015). Roughly 64,000 women are diagnosed with ductal carcinoma *in situ* (DCIS) (stage 0 cancer) annually in the US. Epidemiological studies suggest that about a quarter of these patients have indolent disease, which would not affect a patient during her lifetime (Ozanne et al., 2011; Bleyer and Welch, 2012; Esserman et al., 2013; Marshall, 2014; Harding et al., 2015). Yet all patients are treated as if they have invasive disease. This response seems reasonable as pathologists cannot distinguish indolent from aggressive disease, and both the patients and physicians are gravely concerned about the outcomes. The same concern is found for prostate, lung, thyroid, and other forms of cancer (Esserman et al., 2014). To better understand cellular aggressiveness in biopsy material, we have recently reported biomarker ratio imaging microscopy (BRIM), which can stratify DCIS tissue samples using biomarkers related to cancer aggressiveness and digital image processing. In this protocol article, we provide background information, rationale, and definitive methods with step-by-step instructions to examine FFPE samples using BRIM.

### Background: conventional and unconventional histology

**Figure 1:**
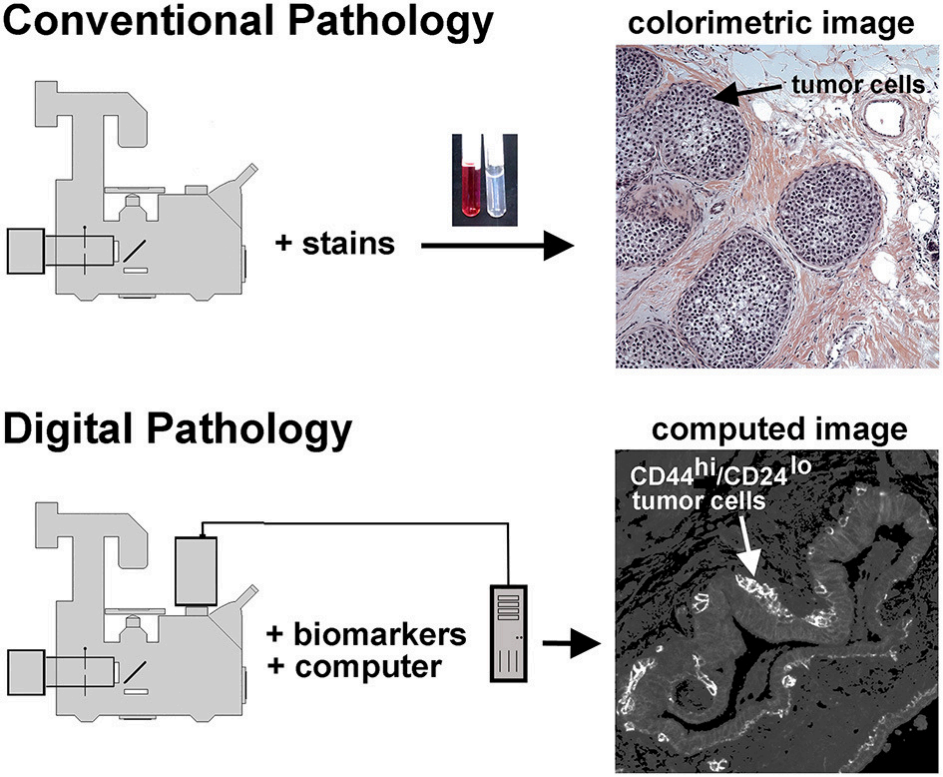
**Conventional and unconventional approaches in tissue evaluation are shown**. As illustrated at the top of this figure, tissues may be stained with dyes to reveal structural features, as illustrated in the H&E stained tissue sample on the right hand side. This image shows a DCIS lesion exhibiting the solid architecture. On the lower portion of this figure we illustrate the digital pathology approach used in the procedures described below. In this case separate images of CD44 and CD24 immunofluorescence are collected, then ratioed *in silico* to reveal a computed image wherein the presence of CD44^hi^/CD24^lo^ cells are highlighted. Thus, a subpopulation of tumor cells can be identified. In this case, an invasive breast disease was studied. Note the presence of a subset of highly positive CD44^hi^/CD24^lo^ cells at the periphery of the duct.

Microscopy has many advantages in studying breast lesions. It permits the detection of early disease because, in principle, single dangerous cells can be imaged. In contrast to cell extraction methods that dilute biomarkers, microscopy allows biomarker detection within cell organelles at high concentrations. Pathology labs also employ microscopy, where fixed tissues embedded in paraffin blocks are standard. Conventional stains, such as hematoxylin and eosin (H&E), provide the necessary resolution to distinguish among structural elements of a tissue, which is exactly what they were intended to do (Figure 1). Biomarkers, such as the estrogen receptor, progesterone receptor and HER-2 are used to evaluate breast cancer tissue sections with immunohistochemistry to provide further information for patient management (Allred, 2010). For many years, it has not yet been possible to use molecularly defined biomarkers to stratify DCIS samples with a sufficient range to provide prognostic information sufficient to identify aggressive vs. non-aggressive lesions. We have recently introduced the BRIM technique, which provides substantially improved stratification of DCIS lesions by using a combination of biomarkers, quantitative microscopy and digital image processing (Clark and Petty, 2016), which should improve our understanding of tumor cell heterogeneity and contribute to patient management.

**Figure 2:**
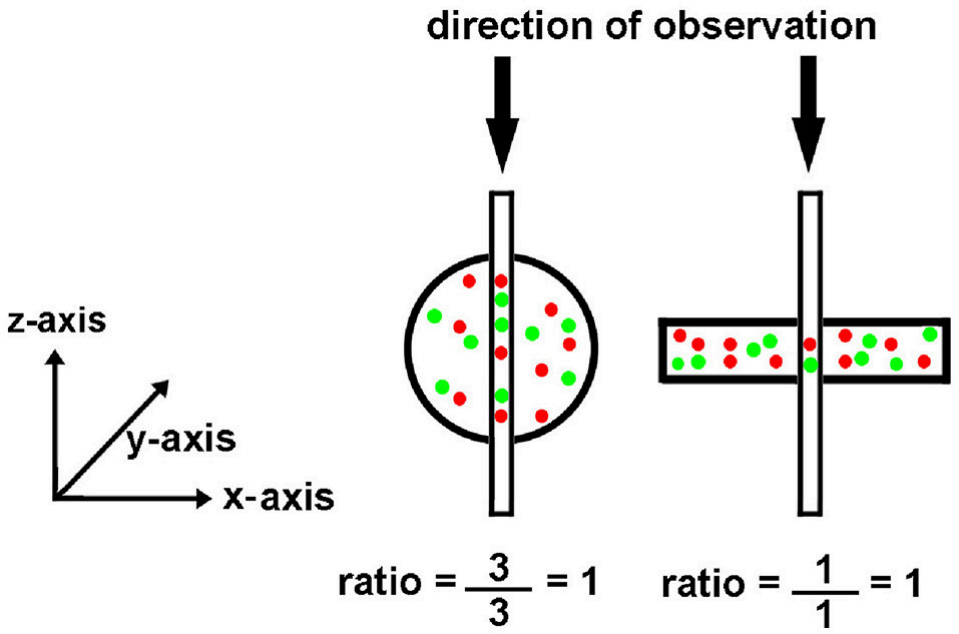
**Effect of object shape on perceived brightness by microscopy**. The circle and rectangle have the same volume and particle number. The circle appears brighter to an observer because it is thicker. The narrow vertical rectangle represents a vertical volume along the line of sight. Although the round cell appears “brighter,” the ratio of red to green tags is the same.

Digital image processing (Figure 1) offers an alternative approach to conventional optical microscopy; during BRIM, at least two fluorescence images are collected then processed by computer. Conventional histology has several drawbacks, as outlined in Table 1. For quantitative studies, the signal intensity must be linear with respect to biomarker number. Hence, enzyme-linked amplification in immunohistochemistry is inappropriate due to its non-linear characteristics (enzyme kinetics, inner filter effects, substrate nucleation, etc.) (Exbrayat, 2013). These difficulties are avoided by fluorescence microscopy. Although fluorescence lamps should always be adjusted for Köhler illumination, it is possible that spatial variations in illumination may be found, which distort image brightness in conventional microscopy. However, ratio imaging microscopy corrects for spatial artifacts in illumination by comparing the same pixel at two different wavelengths. One theoretical limitation of ratio imaging is that the shot noise of a conventional image is $\sqrt{\text{N}}$ (N = the number of counts), whereas the shot noise in a simple ratio image is $\sqrt{2\text{N}}$, as expected by the propagation of error. However, this physical limitation is unimportant in this application because the increase in percent error introduced by ratioing at a bin intensity of 10^4^ counts is only 0.4%, which is far smaller than the biological variability. Variations in section thickness, cell shape, and cell size influence a sample's perceived brightness (Figure 2), but cancel out in ratio imaging microscopy (Bright et al., 1989; O'Connor and Silver, 2007; Petty, 2007). In addition, image ratioing has the distinct advantage of canceling out instrumental factors affecting the brightness of an image including the numerical aperture (na), magnification (mag), transmittance of optics (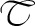
_optics_), the detector's quantum efficiency (D_QE_), the detector's gain (D_gain_), the integration time (t), the fluorophore's quantum efficiency (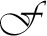
_QE_), and the pathlength (*p*_*l*_). As these instrument-specific elements cancel out, standardization of results among laboratories will be improved.

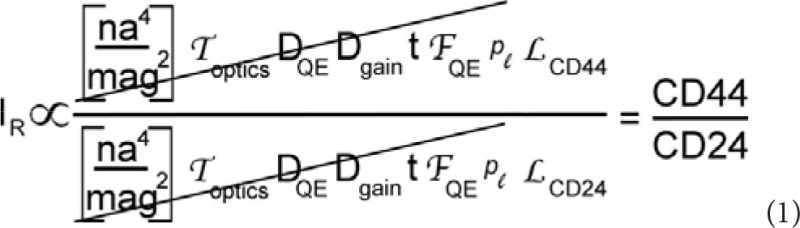



**Table 1:** **Advantages of BRIM Over Conventional Histochemistry**.

**Numbers**	**Drawbacks of conventional imaging**	**BRIM improvements**
1)	variations in cell shape	ratioing
2)	variations in section thickness and loss of cell material during processing	ratioing
3)	non-uniform illumination	ratioing
4)	enzyme activity and non-linear deposition of reaction product	fluorescence
5)	light absorption by product is non-linear	fluorescence
6)	modest optical resolution of reaction products	fluorescence

Image ratioing is best known as a tool to detect calcium signals (Bright et al., 1989; O'Connor and Silver, 2007; Petty, 2007). However, it has also be used to detect pH and membrane potential changes within cells (Bright et al., 1989; O'Connor and Silver, 2007; Petty, 2007). It is also used, although less often, in protein activation, polarization, viscosity, proximity, and water permeability studies (Axelrod, 1989; Nalbant et al., 2004; Doná et al., 2013). This method has also been used to assess the interstitial pH of tumors *in vivo* (Helmlinger et al., 1997). As described above, two images are collected during ratioing at two different wavelengths. As the signal of interest, such as calcium concentration, increases, the image at one wavelength will increase while the image at a second wavelength will decrease in brightness. Although the use of one probe emitting at two wavelengths is frequently used for ratio imaging microscopy, two fluorescent labels are also used in image ratioing (Floto et al., 1995; Clark and Petty, 2008; Clark et al., 2010).

Let us next consider a biomarker thought experiment to illustrate our approach. Previous studies have shown that CD74, the γ chain of HLA class II antigens, increases during invasive breast cancer (Porter et al., 2003; Metodieva et al., 2013). On the other hand, CD59, a complement regulatory protein (Madjd et al., 2003; Porter et al., 2003), decreases in invasive breast cancer. Thus, CD74 expression correlates with poor outcomes while the CD59 level anti-correlates with poor outcomes. As breast cancer is believed to represent a continuum from normal to DCIS to invasive and metastatic disease, we quantitatively compare normal tissue to invasive tissue to estimate the dynamic range in contrast that might be obtained by ratioing experiments. Using the gene expression data of Porter et al. (2003), we find that the average levels of CD74 and CD59 on normal cells were 20 and 41, respectively. In the case of invasive breast cancers, the levels of CD74 and CD59 were 254 and 5. Thus, the ratio of these two parameters on normal cells is expected to be 0.49 whereas the ratio on invasive cells is 50.8. The range of ratios is expected to be 100-fold. As ratioing cancels out path-length and other artifacts, as well as systematic noise in the tissue section, the large potential range in contrast with reduced noise and artifacts allow us to identify CD74^hi^/CD59^lo^ cells in a subpopulation of DCIS patient samples (Clark and Petty, 2016).

Biomarker ratio imaging microscopy (BRIM) may be used to detect well-known cell subpopulations using FFPE samples. As illustrated on the lower right hand side of Figure 1, BRIM can identify specific cell subtypes, such as CD44^hi^/CD24^lo^ cells. Cells structurally defined as CD44^hi^/CD24^lo^ cells have been functionally identified as breast cancer stem cells (Al-Hajj et al., 2003). Similarly, when cells switch from an epithelial (N-cad^lo^/E-cad^hi^ cells) to mesenchymal phenotype (N-cad^hi^/E-cad^lo^ cells), the expression of E-cadherin dramatically declines while the expression of N-cadherin increases. As breast cancer stem cells have been linked to tumor growth and resistance to chemotherapy and radiotherapy (Dean et al., 2005; Li et al., 2008), the presence of CD44^hi^/CD24^lo^ cells in a tissue sample indicates tumor aggressiveness. Similarly, the presence of mesenchymal tumor cells indicates an ability to metastasize (Thiery, 2002; Ledford, 2011; May et al., 2011), which also indicates aggressive tumor cells. Thus, multiple breast cancer cell properties may be assessed using BRIM.

## Materials and equipment

### Equipment

Biomarker ratio imaging microscopy (BRIM) requires a high-sensitivity wide-field fluorescence research microscope, such as the Nikon TE-2000 Quantum, which is used by this laboratory. To maximize light collection, a high numerical aperture objective is used. For this application, we typically employ a 20x/0.5 objective to evaluate tissues, which provides good brightness. Indeed, the mathematic relationship between magnification and numerical aperture (Equation 1) indicates that this Nikon objective provides somewhat brighter images than its 40x/0.6 counterpart. In addition to its brightness, this objective is also a good choice due to: (1) its utility in pathology and (2) at this magnification many fluorescent labels can be found in each pixel thus providing a reliable estimate of the ratio. The optical filter sets require a high % transmittance in the passband, while also providing the greatest out-of-band reflectance. For ratio imaging, filter sets with zero pixel shift image registration should be used (although small registration errors can be corrected in software). For this application, we recommend the Chroma 49000 ET series of optical filters (Chroma Technology Corp., Bellows Falls, VT), which have improved performance characteristics in comparison to conventional optical filters used in fluorescence microscopy. The increased transmittance of these filter sets may enhance the photobleaching of fluorochromes, but this can be managed using embedding media that reduce photobleaching (see below). Specifically, we employ a Chroma 49011 filter set for Alexa Fluor® 488 and a Chroma 49004 filter set for Alexa Fluor® 568. As the emission spectrum of Alexa Fluor® 488 overlaps the excitation spectrum of Alexa Fluor® 568, it is possible that resonance energy transfer between these two labels could artifactually lower the apparent level of Alexa Fluor® 488 emission. This possibility was checked experimentally, and no significant level of RET was observed between labels. This result was expected because the biomarkers are not known to be physically associated with one another. This confirms the appropriateness of the equipment and labels used in these studies.

It is important to choose a camera with high sensitivity, low noise, with a linear response to illumination intensity. As we use a 20x objective, lateral resolution is not important. For this application, back-illuminated electron-multiplying charge coupled device (EMCCD) cameras are appropriate. EMCCD cameras from Andor Technology (Belfast, UK) and Photometrics (Tucson, AR) are good choices. These ultra-sensitive cameras can: detect roughly 1 to 10 photons, operate at low noise (the EMCCD chips are cooled to −80°C), and have high quantum efficiencies with outputs that are linearly proportional with illumination intensity. As these cameras are built for biological imaging, their spectral responsivities are nearly 100% in the green-to-red region of the spectrum, but decrease at higher and lower wavelengths. The Andor iXon DV887 camera has 16 × 16 mm pixels providing a 220,000 pixel well depth. As ratioing assumes that the images can be quantitatively compared, it is essential to confirm the linearity of the camera output, as described in the next section.

### Verification of camera linearity using bead intensity calibration

An InSpeck microscopy image intensity calibration kit (ThermoFisher Scientific/Molecular Probes) was used to calibrate the detector. These beads possess different fluorescence intensities. Beads with 0, 0.4, 1.3, and 4.2% fluorescence were diluted and imaged with the apparatus described above. Images of blue fluorescent beads were taken using excitation filter of 350/50 nm, a 415 DRLP dichroic reflector, and a 450DF30 nm emission filter. Camera settings were identical to those used in all experiments (see below). It is important to select a range of bead fluorescence intensities that include the range of fluorescence intensities to be encountered with biomarker labeling.

**Figure 3:**
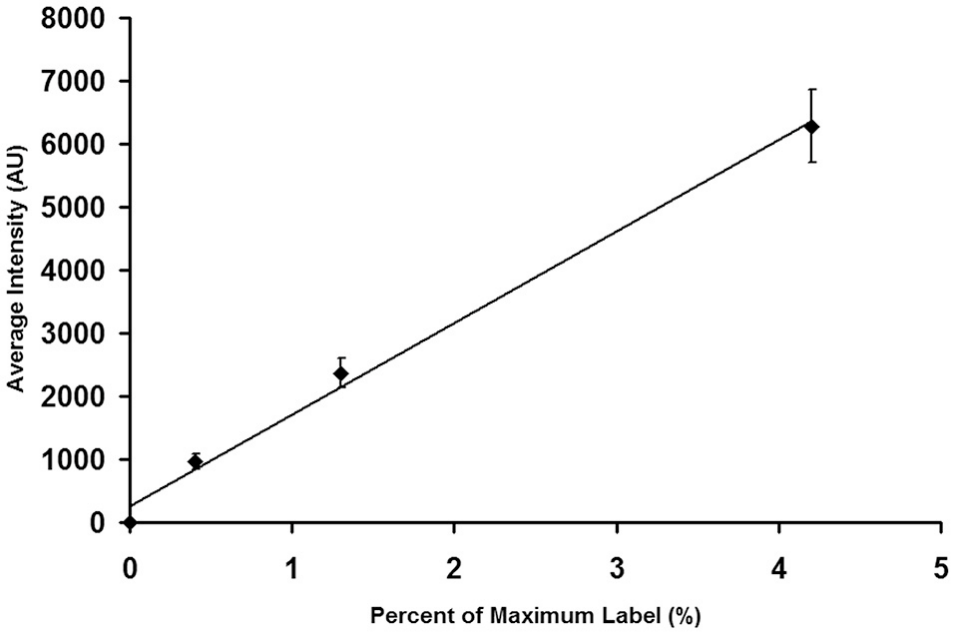
**Calibration of an EMCCD Camera**. It is important to calibrate the camera output to insure that its performance is linear throughout the brightness region studied in tissue experiments. As described in the text, individual beads were photographed. The images were then analyzed to determine the average intensity of each bead preparation. The average bead intensity is plotted at the ordinate and the percentage of maximal label, as provided by the manufacturer, is plotted at the abscissa. Note that the output of the camera is linear with the extent of label.

After saving the images, they were opened in MetaMorph and spherical regions of interest were drawn over the beads. Region measurements were performed to collect average intensity readings for each type of bead. Measurements were imported into Microsoft Excel, where the average intensity and standard deviation were calculated for each bead type. Intensity averages were plotted vs. the percent of relative intensity given for each bead type in the kit. The standard deviation for each point was added as y-error bars, and a linear trend line was calculated. Figure 3 shows a calibration curve for the camera output, which was found to be linear.

### Supplies

The chemical supplies for BRIM experiments are listed in Table 2. The antibodies used in these studies are given in Table 3. Alexa Fluor® fluorescence labels are used because of their brightness and photostability. The software programs used in these studies are Metamorph (v. 7.1.2.0) (Molecular Devices), Metafluor (v. 7.1.2.0) (Molecular Devices), and ImageJ (NIH).

**Table 2:** **Supplies List for BRIM Protocol**.

phosphate buffered saline tablets (Life Technologies; cat# 003002)
Triton X-100 (Sigma; cat# T8787-100 mL)
o-xylene (Sigma; cat# 295884-2 L)
Tween® 20 (Sigma; cat# P7949-100 mL)
citric acid (Sigma; cat# C2404)
sodium citrate dihydrate (Sigma; cat# W302600)
albumin from bovine serum–BSA (Sigma; cat# A7906-50 g)
non-fat dried milk
Prolong Diamond anti-fade mountant (ThermoFisher; cat# P36961)
corning cover glass no. 1 thickness (Corning; 18 × 18mm cat# 2845–18, 25 × 25mm cat# 2865–25, 24 × 50mm cat# 2975–245)
super PAP pen (ThermoFisher; cat# 008899)


**Table 3:** **Antibody List for BRIM Protocol**.

**PRIMARY**				
**Antibody**	**Source**	**Cat #**	**Species**	**Ratio**
N-Cadherin	Abcam	ab98952	Ms	N-Cad/E-Cad
E-Cadherin	Abcam	ab15148	Rb	N-Cad/E-Cad
CD 74	Abcam	ab9514	Ms	CD74/CD59
CD 59	Abcam	ab133707	Rb	CD74/CD59
CD 44	Abcam	ab41478	Rb	CD44/CD24
CD 24	Biolegend	311102	Ms	CD44/CD24
**SECONDARY**				
**Antibody**	** Source**		** Cat #**	** Species**
Gt anti-Ms Alexa 488	Invitrogen		A11029	Gt
Dk anti-Rb Alexa 568	Invitrogen		A10042	Dk

### Biomarkers

Although numerous biomarkers have been identified, they are not necessarily useful in BRIM studies. The labeling intensity needs to be appropriate: either rising or falling with aggressiveness sufficient to provide a high dynamic range after ratioing. The biomarker distributions within cells are also important. We have found that ribosomal biomarkers are too punctate to ratio. We have also found that biomarkers, such as gene regulatory proteins are not generally useful in BRIM studies. These biomarkers can translate between the cytoplasm and the nucleus and cannot be reliably used with current software. Thus, the biomarker pairs must be in the same cell locations for BRIM experiments. We have found that plasma membranes or mitochondria are particularly useful targets for BRIM experiments.

### Samples

Formalin fixed paraffin-embedded (FFPE) tissue samples were obtained from National Disease Research Interchange (Bethesda, MD), the Cooperative Human Tissue Network (Columbus, OH), and Fox Chase Cancer Center (Philadelphia, PA). These studies were approved by the University of Michigan's IRB with informed consent in accordance with the Declaration of Helsinki.

## Step-by-step procedures

### Paraffin removal and antigen retrieval

Formalin fixed paraffin-embedded (FFPE) samples are routinely used by pathology laboratories, and fit within their normal workflows. We have used BRIM to evaluate FFPE samples after over 10 years of storage. Others have reported that these immunochemical procedures can be successfully employed to study biomarkers on samples after 30 years of storage (Camp et al., 2000). Hence, BRIM should be widely useful in the evaluation of pathology samples.

Paraffin Removal:

1. Obtain paraffin sections from FFPE tissue blocks, and then mount them on slides.
2. Heat sections to 60°C in a dry oven for 1 h to soften paraffin.
3. Wash each slide twice in xylene for 5 min to remove paraffin.

Rehydration via ethanol gradient:

4. Wash slides two times for 5 min each in 100% ethanol,
5. Wash for 1 min in each of the following solutions: 95, 80, and 70% of ethanol in water.
6. Wash slides in phosphate buffered saline (PBS) containing 0.02% Triton X-100 two times for 5 min each.

Antigen Retrieval:

7. Transfer slides to a slide rack and place in a beaker containing antigen retrieval buffer (10 mM citrate buffer at pH 6.0 with 0.05% Tween® 20).
8. Place the beaker containing racked slides in a pot of boiling water, cover with a lid, and steam for 10 min.
9. Remove the beaker and allow it to cool to room temperature for 1 h.

### Antibody labeling of sections

It is possible that different antibodies directed against the same biomarker may react with tissue antigens in ways that affect the ratio observed. This could be due to binding differences, epitope differences, antigen retrieval differences, or other factors. Hence, it is crucial to use antibodies specifically reported for this application (Table 3).

Preparation for Antibody Labeling:

1. Wash slides three times for 15 min each in PBS containing 0.02% Triton X-100.
2. Remove Triton X-100 detergent by washing three times for 15 min each in PBS.
3. Remove slides from wash and dab off excess PBS; circle tissue using a hydrophobic PAP pen to facilitate labeling.
4. Cover tissue with a solution of 10% non-fat dried milk in PBS to block non-specific binding and then place onto a humidified tray for 1 h at room temperature.

Primary Antibody Labeling:

5. After blocking for 1 h, remove the solution and briefly rinse the slide by dipping into PBS.
6. Cover tissue section with appropriate Ms and Rb primary antibodies at 2 μg/mL (usually 1:100) in PBS containing 1% BSA.
7. Gently place slides on a humidified tray that is kept overnight at 4°C.

Secondary Antibody Labeling:

8. Wash slides three times for 15 min each in PBS, to remove unbound primary antibodies.
9. Dab off excess PBS, and cover the tissue section with secondary antibodies [Alexa Fluor® 488 goat anti-mouse (Invitrogen A11029) and Alexa Fluor® 568 Donkey anti-rabbit (Invitrogen A10042)] at 20 μg/mL each, in PBS containing 1% BSA.
10. Place slides on a humidified tray, and move them to the dark for 1 h, at room temperature.

Mounting Slides:

11. Wash slides three times for 15 min each in PBS, in the dark, to remove unbound secondary antibodies.
12. Remove slides individually, and dab off excess PBS.
13. Place 1–2 drops (depending on section size) of ProLong® Diamond antifade mountant (ThermoFisher Scientific / Molecular Probes, Eugene, OR) on one end of the section.
14. Place one side of an appropriately-sized coverslip against the drop(s) of mountant and then slowly and carefully lower the coverslip using a pipette tip for support to prevent bubbles from forming.
15. Allow slides to cure at room temperature in the dark for at least 12 h prior to imaging.

### Microscopy

1. Image slides using a Nikon 20x/0.5 Plan Fluor objective on a Nikon TE-2000 inverted microscope via an Andor iXon camera (model DV887ECS-BV) attached to the bottom port. Plan Fluor or Super Fluor objectives are recommended due to their chromatic correction, high numerical aperture, transmittance and flatness across the field.
2. Adjust the Hg lamp for Köhler illumination and then minimize the iris diameter outside the camera's field of view. This procedure reduces the entry of stray fluorescence light from outside the field of view into the objective.
3. Conduct an initial evaluation of the sample to locate an area of the section where the numerator biomarker shows bright staining for imaging.
4. Acquire images via MetaMorph software with the following settings: exposure time = 100 ms; average = 10 images; EM gain = 100; CCD temperature = −80°C; 14-bit digitizer; vertical shift speed = 294.12 kHz; vertical clock voltage = normal; pre-amplifier gain = 1.00 x; camera state = non-overlapped; binning = 1.
5. Take a white light image of the field without EM gain for reference.
6. Collect a green fluorescence image using a Chroma 49011 filter set comprised of a 480/40 nm excitation filter, a 510 nm dichroic reflector, and a 535/50 nm emission filter.
7. Switch the filter cube to a Chroma 49004 filter set comprised of a 545/25 nm excitation filter, a 535 nm dichroic reflector, and a 605/70 nm emission filter to collect an image of red fluorescence. Both images are collected of the same microscopic field at the same focal plane.
8. Save and then close images.

### Image processing

1. Reopen biomarker image pairs (Alexa Fluor® 488 and 568) of the same field in MetaMorph software.
2. Use the “add plane” function to create a two image stack: the image corresponding to the numerator should be on top of the stack whereas the image corresponding to the denominator should be on the bottom of the stack.
3. Save and then close the image stack.
4. Open MetaFluor software, and select the “build INF file” option under the utilities tab.
5. Select each image stack from the previous step and make an.INF file using the following options: Image type–Stack file; number of wavelengths–2; equally space images by 102 ms (image spacing is unnecessary); the files can now be opened by MetaFluor.
6. Open the.INF files in the MetaFluor workspace, which will result in a ratio image. Select “display” to allow the image parameters to be set, including the range of the calculated ratio image displayed (in this case 0–4). An exclusive threshold should be set on each of the original images to eliminate areas of the image that are “holes” in the section itself (no cells or tissue).
7. Select the “Save ratios” option to name the ratio image file. File name includes sample identification code, the field number, ratio range, and the antigens used in the ratio. Press the play button to save the image.

### Image analysis: quantification of image data

Although images can be judged as high or low BRIM tissue samples by simple visual inspection, it is necessary to quantify image properties for statistical analysis.

1. Use ImageJ software to further process the raw ratio tiff files. After opening, confirm that the Image Type on the pull-down menu is 8-bit.
2. On the Image-Adjust-Threshold pop-up window, adjust the count area to 130–255. As previously described (Clark and Petty, 2016), this range of values was selected because it corresponded to ratios uniquely associated with aggressive disease. Investigators may need to assess the proper lower bound for their particular experimental set-up to avoid overlap with normal breast tissue or fibroadenoma tissue (Clark and Petty, 2016). This threshold removes essentially all of the low ratios associated with normal tissue; if the red button is pressed, the ratios associated with aggressive biomarker ratios will be highlighted red in the image.
3. Under the Analyze menu, depressing the Analyze Particles command of the pull-down bar will cause the analyze particles pop-up window to be shown. Type in 5 for the minimum size of the particles in pixels. Smaller particle sizes may have a tendency to pick up noise in the ratio image. This step produces several image metrics, such as count and area. Parameters, such as particle count can be used to compare patient samples.

## Results and discussion

### Suppression of systematic noise and pathlength differences

**Figure 4:**
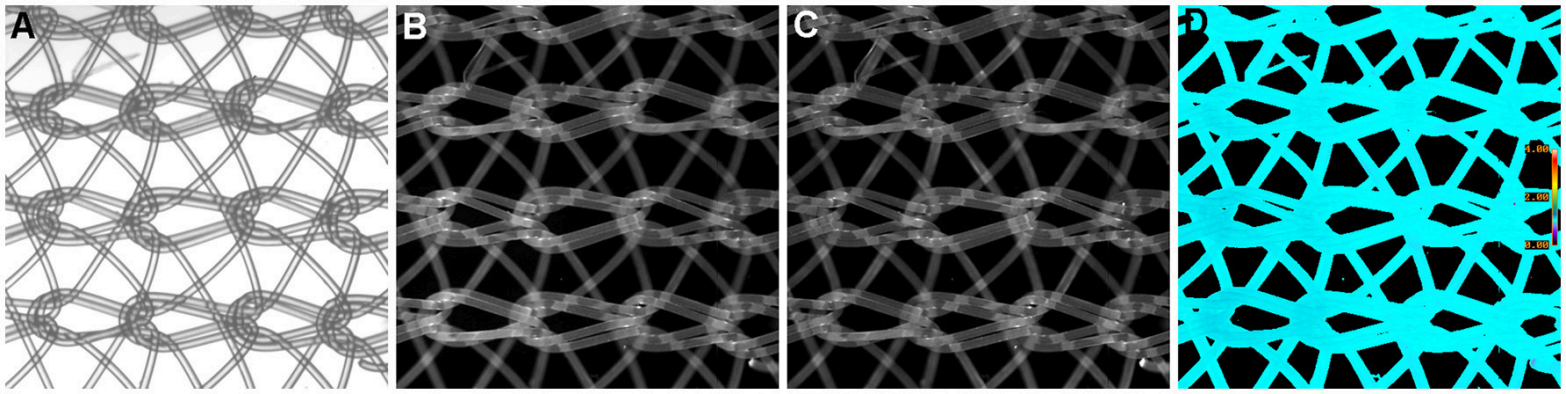
**Example of Ratio Imaging Microscopy**. A nylon mesh was non-specifically coated with Alexa Fluor® 488-labeled antibodies and Alexa Fluor® 568-labeled antibodies then air-dried. **(A)** shows the meshwork using bright-field microscopy; the overlapping regions should be noted. **(B,C)** show the green and red fluorescence emission channels, respectively. The ratio image of Alexa Fluor® 488 emission divided by Alexa Fluor® 568 emission was calculated, which is shown in **(D)**. It should be noted that the pathlength differences in areas of mesh overlap disappear and that the non-specific staining in **(B,C)** disappear by ratioing to one. (Ratio scale bars are given along the right hand side of each panel.) (5x objective).

We have previously mentioned some of the advantages of ratio imaging microscopy in the analysis of tissue sections (Table 1, Equation 1). Systematic noise is a common element in conventional histology. For example, in our recent paper (Clark and Petty, 2016), we illustrated the suppression of systematic noise during BRIM. Irregularities in sections, such as knife chatter, is suppressed because it is present in both the numerator and denominator images, and hence, cancels out when the ratio is computed. Similar types of systematic noise, such as scratches due to machining of slide or cover-slip surfaces or patterns formed when nylon or formvar matrices are used as support structures should be minimized. Although the non-specific binding of antibodies can be managed by blocking reagents, it is not perfect. The non-specific staining of tissues with fluorescent second-step antibodies is a potential source of systematic noise. When this idea was tested in a BRIM experiment (with the brightness increased to make the non-specific fluorescence easier to visualize), we found that the non-specific component of sample fluorescence was dramatically reduced (data not shown). In Figure 4, we show a mesh support with non-specifically bound Alexa Fluor® 488-labeled antibodies and Alexa Fluor® 568-labeled antibodies. Areas of mesh overlap are brighter because they are thicker than regions of single strands. However, after ratioing, the non-specific staining of the mesh disappears because it is ratioed to one (Figure 4D); this also illustrated that the pathlength artifacts of overlapping strands also disappear. This approach may be useful in the analysis of tissues with high levels of autofluorescence. For example, lipofusin in tissues, such as the retina is highly fluorescent across the visible spectrum, and could be managed with this approach. Thus, the methodology described above might be applicable in many areas of pathology.

### Illustration of BRIM

**Figure 5:**
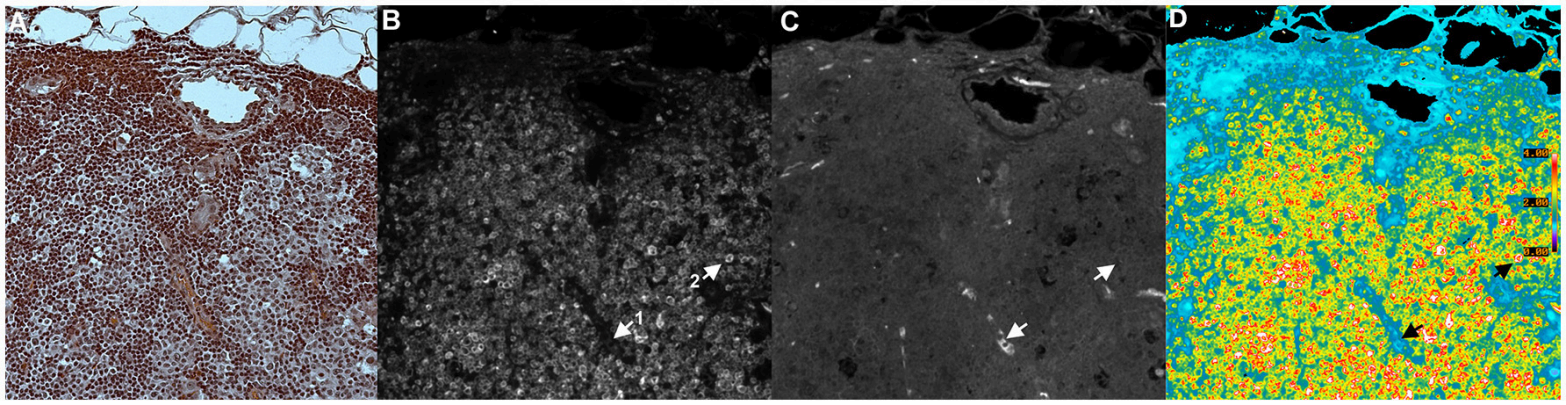
**Tissue Illustration of BRIM**. This figure shows images of: **(A)** H and E image, **(B)** CD74 (numerator), **(C)** CD59 (denominator), and **(D)** CD74/CD59 (ratio). The micrograph in **(A)** was obtained using H&F staining followed by imaging with a color camera. This tissue sample was derived from a patient diagnosed with invasive breast cancer. As H&F staining should not be performed on the same slide as immunofluorescence imaging, the micrographs of **(A–C)** were taken from serial sections. Hence, **(A)** is similar to, but not identical with, **(B–D)**. The images of **(B,C)** were autoscaled for clarity of illustration, but not in the calculation of the ratio in **(D)**. This image was chosen for pedagogical purposes because it illustrates the principles of BRIM. Due to the brightness in each channel, cell subtypes are easily discerned. **(B)** shows the numerator, CD74. Two cells are designated as 1 and 2. Cell 1 is a CD74^lo^/CD59^hi^ cell whereas Cell 2 is a CD74^hi^/CD59^lo^ cell. This illustrates the fact that different cell populations are present. As the differences in CD74 and CD59 levels are very large, a considerable improvement in gain can be observed. As anticipated by genomic studies (Porter et al., 2003), large differences in CD74 and CD59 levels can be observed in invasive breast cancer cells, as physically manifested in the BRIM micrograph of **(D)**. In the case of DCIS, a large range of ratios is observed (Clark and Petty, 2016). **(D)** shows a field containing many CD74^hi^/CD59^lo^ cells. Such high numbers of CD74^hi^/CD59^lo^ cells are not found in normal breast tissue, fibroadenoma of the breast, and a sub-population of DCIS samples (Clark and Petty, 2016). A ratio scale bar is given along the right hand side of **(D)** (20x objective).

**Figure 6:**
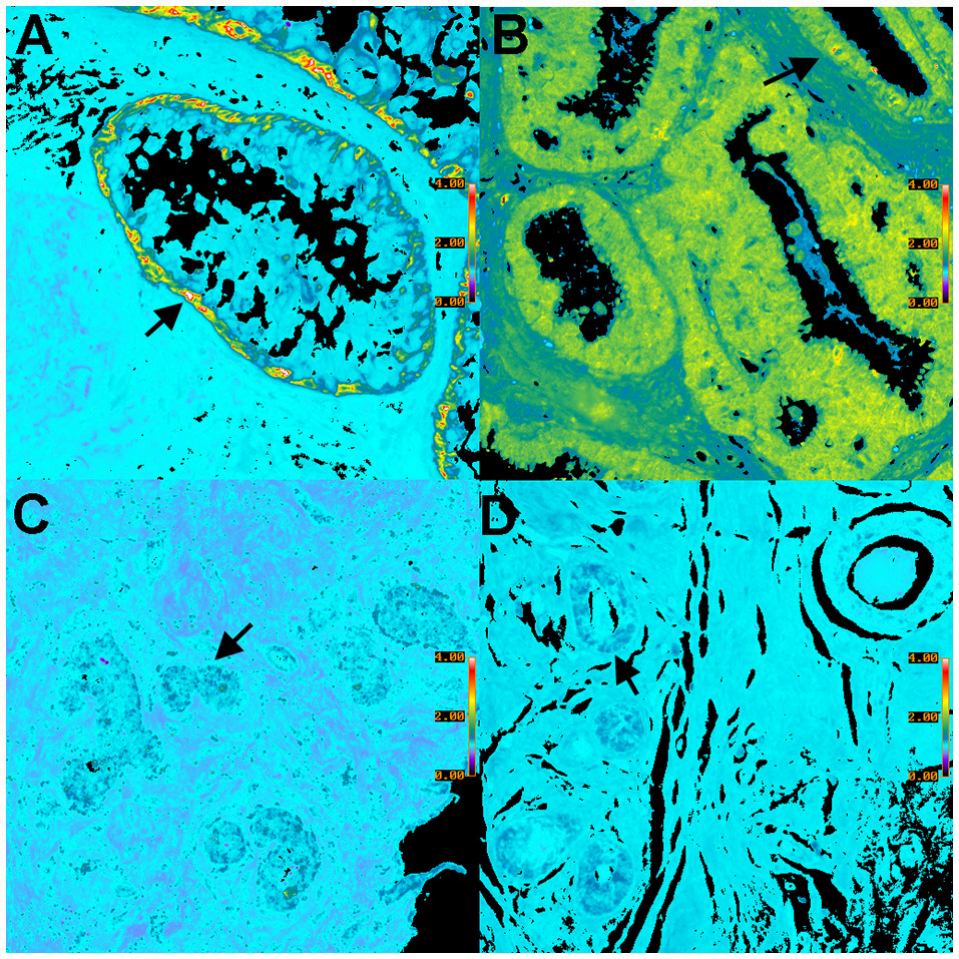
**Pseudocolor BRIM images of CD44^**hi**^/CD24^**lo**^ cells and N-cad^**hi**^/E-cad^**lo**^ cells of DCIS lesions are shown. (A,C)** show CD44^hi^/CD24^lo^ cells whereas **(B,D)** show N-cad^hi^/E-cad^lo^ cells. Positive BRIM findings (high ratios) are shown in **(A,B)**. Negative BRIM results (low ratios) are shown in **(C,D)**. Higher ratios are indicated by the red-yellow colors in **(A,B)**. Arrows denote regions of higher ratios. Note that the CD44^hi^/CD24^lo^ cells are primarily found at the periphery of the ducts in **(A)**. These data show that patients can differ widely in the ratios associated with DCIS. (Ratio scale bars are given along the right hand side of each panel) (20x objective).

Figure 5 shows an example of a BRIM experiment. These micrographs show CD74 and CD59 experiments (examples of N-cadherin, E-cadherin, CD44 and CD24 experiments are shown below in Figure 6). Figure 5A shows an H and E image of a serial section of this region of the sample. CD74 (numerator) and CD59 (denominator) are shown in Figures 5B,C, respectively. A pseudocolor ratio image is shown in Figure 5D. In these three micrographs, a CD74^lo^/CD59^hi^ cell and a CD74^hi^/CD59^lo^ cell are illustrated as cell 1 and 2, respectively. Thus, when CD74 is high and CD59 is low, very large ratios are observed. Alternatively, when CD74 is low and CD59 is high, very low ratios are found. As Figure 5D shows, a wide range of ratios are obtained. Importantly, many CD74^hi^/CD59^lo^ cells are found, which suggests that the lesion contains aggressive cells, as CD74 correlates with adverse patient outcomes and CD59 correlates with positive patient outcomes (or inversely correlates with aggressiveness) (Madjd et al., 2003; Porter et al., 2003; Metodieva et al., 2013). The high number of CD74^hi^/CD59^lo^ cells is consistent with the diagnosis of invasive breast cancer.

### Characterization of cell types in DCIS

We will now illustrate the results obtained when BRIM is used to study DCIS lesions. Figure 6 shows BRIM micrographs of DCIS lesions stained for CD44^hi^/CD24^lo^ cells and N-cad^hi^/E-cad^lo^ cells. Figure 6A shows that CD44^hi^/CD24^lo^ cells, which are a key feature of breast cancer stem cells (Al-Hajj et al., 2003), may be found in DCIS ducts, especially at the perimeter. However, DCIS lesions of other patients can be negative, as illustrated in Figure 6C. Similarly, Figures 6B,D show N-cad^hi^/E-cad^lo^ cells, which illustrate the mesenchymalness of the cells, a trait associated with the ability of cells to metastasize (Li et al., 2008). Positive cells are shown in panel B, whereas negative cells are shown in panel D. Distributions of BRIM scores of a DCIS patient population for stemness and mesenchymalness is shown in our previous study (Clark and Petty, 2016). Thus, the suppression of noise and the enhancement of BRIM offer significant advantages in the identification of aggressive and non-aggressive cells in DCIS lesions.

### Conclusions and future applications

In this protocol article we have described, in substantial detail, BRIM, a new and unconventional approach in the study of tissue samples that provides unprecedented insight into the aggressiveness of cancerous lesions. To quickly implement these protocols, we recommend collaborations between biophysicists with experience in ratio imaging microscopy, such as the ratio imaging of calcium concentrations, and scientists or physicians interested in immunohistology.

We anticipate that this approach will impact both basic cancer research and in the clinical assessment of biopsies. The many advantages of BRIM could also be extended to other areas of cancer research, such as other types of over-diagnosed cancers (Esserman et al., 2014). As breast cancer stem cells are resistant to chemotherapy and radiotherapy (Dean et al., 2005; Li et al., 2008), the extent of CD44^hi^/CD24^lo^ cells in biopsies may provide information regarding potential level resistance to therapy, and thereby help to guide treatment. BRIM will also find many applications in drug development because of its lower cost than other methods for assessing the phenotypic properties of tumor cells. For example, one could ascertain the phenotype of tumor cells within animal samples after treatment with emerging anti-stem cell or anti-mesenchymal cell pharmaceuticals. In addition, other types of biomarkers could be employed. For example, BRIM could be used to monitor the percentage of a phosphorylated oncogene vs. the total oncogene pool, wherein the anti-oncogene antibody is a “standard candle” to assess relative changes in phosphorylation. This could also be employed more broadly to investigate signal transduction in tissues. Differences in the locations of signal transduction events within tissues and within single cells could be visualized. Hence, we feel that BRIM will be broadly used in the analysis of tissue properties, and that many additional applications will be found.

## Author contributions

AC performed the imaging experiments. HP invented the method, designed the experiments and wrote the manuscript.

### Conflict of interest statement

The authors declare that the research was conducted in the absence of any commercial or financial relationships that could be construed as a potential conflict of interest. The University of Michigan is the owner of a provisional patent on this subject matter.
